# Poly[[diaqua­bis­(μ-oxalato-κ^4^ *O* ^1^,*O* ^2^:*O* ^1′^,*O* ^2′^)bis­(μ_3_-5-oxidopyridin-1-ium-3-carboxyl­ato-κ^3^ *O* ^3^:*O* ^3′^:*O* ^5^)diholmium(III)] dihydrate]

**DOI:** 10.1107/S1600536812032916

**Published:** 2012-08-04

**Authors:** Jun-Long Mi, Jing Huang, Hong-Ji Chen

**Affiliations:** a Department of Materials Science and Engineering, Jinan University, Guangzhou 510632, People’s Republic of China

## Abstract

In the title compound, {[Ho_2_(C_6_H_4_NO_3_)_2_(C_2_O_4_)_2_(H_2_O)_2_]·2H_2_O}_*n*_, the Ho^III^ atom is coordinated by three O atoms from three 5-hy­droxy­nicotinate ligands, four O atoms from two oxalate ligands, each lying on an inversion center, and one water mol­ecule in a distorted square-anti­prismatic geometry. The 5-hy­droxy­nicotinate ligand is protonated at the N atom and deprotonated at the hy­droxy group. The Ho^III^ atoms are bridged by the carboxyl­ate and phenolate O atoms, forming a three-dimensional framework. N—H⋯O and O—H⋯O hydrogen bonds, as well as π–π inter­actions between the pyridine rings [centroid–centroid distance = 3.794 (2) Å], are observed.

## Related literature

For background to the applications of compounds with metal-organic framework structures, see: Allendorf *et al.* (2009 ▶); Choi *et al.* (2008 ▶); Dang *et al.* (2010 ▶); Ishikawa *et al.* (2005 ▶); Lazare *et al.* (2010 ▶); Shimomura *et al.* (2010 ▶); Thallapally *et al.* (2010 ▶). For related structures, see: Zhang *et al.* (2012 ▶).
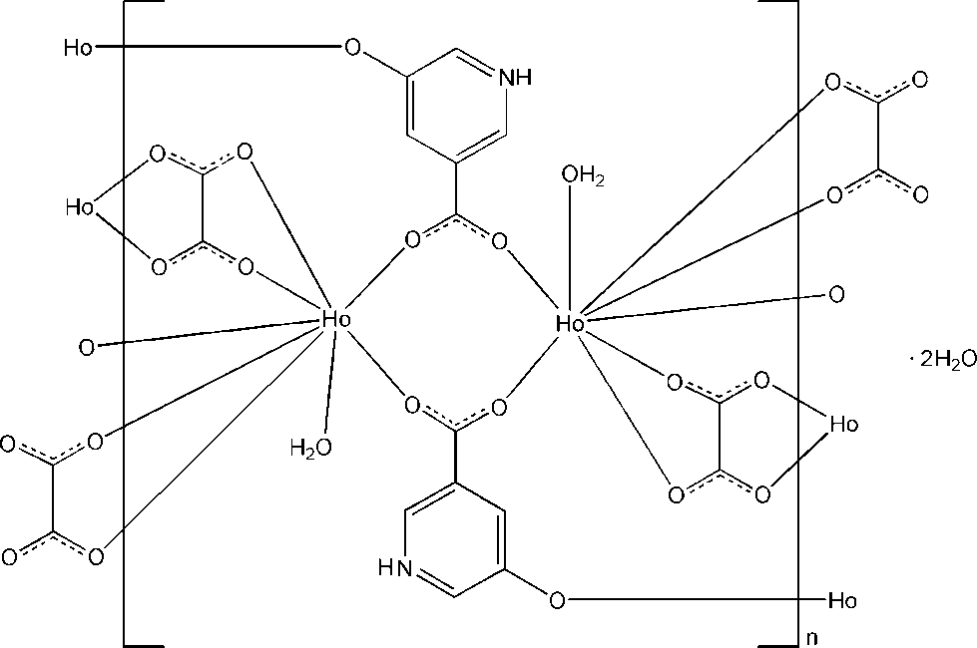


## Experimental

### 

#### Crystal data

[Ho_2_(C_6_H_4_NO_3_)_2_(C_2_O_4_)_2_(H_2_O)_2_]·2H_2_O
*M* *_r_* = 854.16
Triclinic, 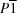

*a* = 7.7786 (16) Å
*b* = 8.0562 (17) Å
*c* = 9.505 (2) Å
α = 110.912 (3)°
β = 96.862 (3)°
γ = 95.770 (3)°
*V* = 545.8 (2) Å^3^
*Z* = 1
Mo *K*α radiation
μ = 7.30 mm^−1^
*T* = 173 K
0.18 × 0.16 × 0.06 mm

#### Data collection

Bruker APEXII CCD diffractometer
Absorption correction: multi-scan (*SADABS*; Sheldrick, 1996 ▶) *T*_min_ = 0.354, *T*_max_ = 0.669
4550 measured reflections
2295 independent reflections
2160 reflections with *I* > 2σ(*I*)
*R*_int_ = 0.017

#### Refinement

*R*[*F* ^2^ > 2σ(*F* ^2^)] = 0.016
*wR*(*F* ^2^) = 0.038
*S* = 1.05
2295 reflections
192 parameters
5 restraints
H atoms treated by a mixture of independent and constrained refinement
Δρ_max_ = 0.57 e Å^−3^
Δρ_min_ = −0.49 e Å^−3^

### 

Data collection: *APEX2* (Bruker, 2007 ▶); cell refinement: *SAINT* (Bruker, 2007 ▶); data reduction: *SAINT*; program(s) used to solve structure: *SHELXTL* (Sheldrick, 2008 ▶); program(s) used to refine structure: *SHELXTL*; molecular graphics: *DIAMOND* (Brandenburg, 1999 ▶); software used to prepare material for publication: *SHELXTL*.

## Supplementary Material

Crystal structure: contains datablock(s) I, global. DOI: 10.1107/S1600536812032916/hy2567sup1.cif

Structure factors: contains datablock(s) I. DOI: 10.1107/S1600536812032916/hy2567Isup2.hkl

Additional supplementary materials:  crystallographic information; 3D view; checkCIF report

## Figures and Tables

**Table 1:** Hydrogen-bond geometry (Å, °)

*D*—H⋯*A*	*D*—H	H⋯*A*	*D*⋯*A*	*D*—H⋯*A*
N1—H1⋯O7^i^	0.90 (1)	1.83 (1)	2.727 (3)	171 (3)
O8—H7⋯O9^ii^	0.85 (1)	1.95 (1)	2.787 (4)	171 (5)
O8—H8⋯O4^iii^	0.85 (1)	1.96 (1)	2.811 (3)	177 (6)
O9—H9⋯O3^iv^	0.85 (1)	2.08 (1)	2.929 (4)	178 (6)
O9—H10⋯O1^ii^	0.85 (1)	2.48 (6)	3.003 (4)	120 (5)

Symmetry codes: (i) 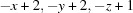; (ii) 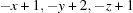; (iii) 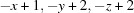; (iv) 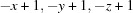.

**Fig. 1.**
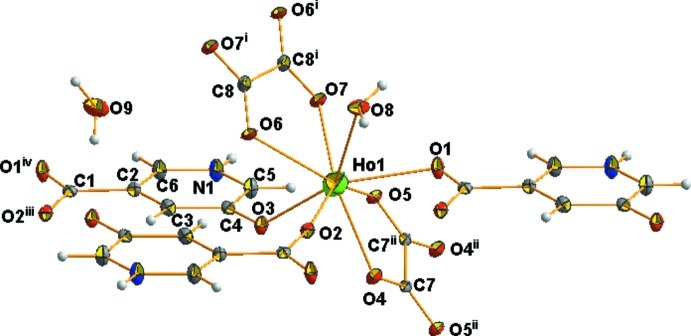
The asymmetric unit of the title compound, showing displacement ellipsoids at the 50% probability level. [Symmetry codes: (i) 1-x, 2-y, 1-z; (ii) 2-x, 2-y, 2-z; (iii) 1-x, 1-y, 1-z; (iv) x, -1+y, -1+z.]

**Fig. 2.**
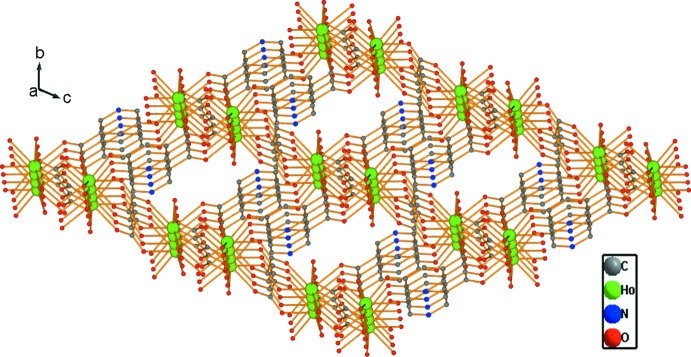
The three-dimensional framework of the title compound. H atoms and solvent water molecules are omitted for clarity.

**Fig. 3.**
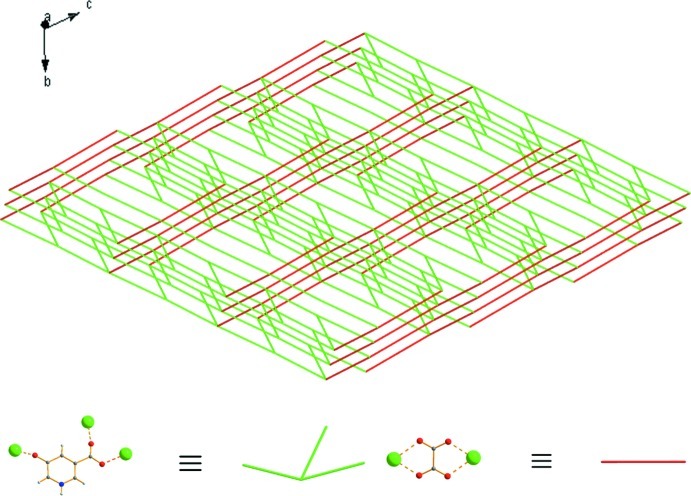
A schematic view of the three-dimensional framework for the title compound.

## supplementary crystallographic information

### Comment

Metal-organic frameworks (MOFs) remain nowadays one of the most studied topics in synthetic chemistry due to MOFs hold interesting structural features and properties. For example, they can be employed as effective heterogeneous catalysts (Dang *et al.*, 2010; Lazare *et al.*, 2010; Thallapally *et al.*, 2010), selective adsorption of gases (Choi *et al.*, 2008; Shimomura *et al.*, 2010), photoluminescent (Allendorf *et al.*, 2009) and magnetic properties (Ishikawa *et al.*, 2005).

The title compound is isostructural with its Dy(III) and Er(III) analogues (Zhang *et al.*, 2012). As shown in Fig. 1, the asymmetric unit is composed of one Ho^III^ atom, one phenoxonicotinate ligand, two halfs of oxalate ligands, one coordinated and one solvent water molecules. The Ho^III^ atom is coordinated by eight O atoms, exhibiting a distorted square-antiprismatic geometry. One basal square face of the antiprism is defined by two carboxylate O atoms, one oxalate O atom and one aqua O atom; the other base is completed by the other three oxalate O atoms and one phenolate O atom. Adjacent Ho^III^ atoms are bonded to the carboxylate and phenolate O atoms of the phenoxonicotinate ligand, forming dinuclear subunits, which are further extented at a *syn-anti* conformation into infinite ladder-like chains. The metal atoms are also bridged by oxalate ligands in a side-by-side manner, forming one-dimensional zigzag chains. Both the ladder-like and zigzag chains are finally linked together through the metal atoms into a three-dimensional framework with one-dimensional microchannels (Fig. 2). The three-dimensional framework shows a topology of 3,5-connected {4^2^.6^5^.8^3^}.{4^2^.6} (Fig. 3). N—H···O and O—H···O hydrogen bonds, as well as π–π interactions between the pyridine rings [centroid–centroid distance = 3.794 (2)Å] are found in the crystal.

### Experimental

A mixture of holmium nitrate (0.4 mmol, 0.181 g), 5-hydroxynicotinic acid (0.8 mmol, 0.111 g), ammonium oxalate (0.8 mmol, 0.099 g) and 10 ml water was sealed in a 15 ml Teflon-lined autoclave. Colorless crystals suitable for X-ray crystallography analysis were obtained by heating the mixture at 453 K for 72 h and then cooled down to room temperature at a rate of 5 K h^-1^ (yield: 41%). Analysis, calculated for C_16_H_16_Ho_2_N_2_O_18_: C 22.50, H 1.89, N 3.28%; found: C 22.65, H 1.91, N 3.13%. IR (cm^-1^, KBr): 3398 s, 3083 s, 2957 m, 2768 w, 2089 w, 1900 w, 1646 m, 1565 m, 1447 w, 1427 w, 1359 s, 1319 s, 1302 s, 1140 s, 1051 s, 1013 s, 963 s, 942 m, 894 w, 865 w, 823 w, 788 w, 672 m, 591 s, 545 s, 497 m, 450 w, 413 w.

### Refinement

H atoms bonded to C atoms were positioned geometrically and refined as riding atoms, with C—H = 0.95 Å and *U*_iso_(H) = 1.2*U*_eq_(C). H atoms bonded to N and O atoms were located from a difference Fourier map and refined isotropically, with distance restraints of N—H = 0.90 (1) and O—H = 0.85 (1) Å.

### Crystal data

**Table:**

[Ho_2_(C_6_H_4_NO_3_)_2_(C_2_O_4_)_2_(H_2_O)_2_]·2H_2_O	*Z* = 1
*M**_r_* = 854.16	*F*(000) = 404
Triclinic, *P*1	*D*_x_ = 2.599 Mg m^−^^3^
Hall symbol: -P 1	Mo *K*α radiation, λ = 0.71073 Å
*a* = 7.7786 (16) Å	Cell parameters from 4550 reflections
*b* = 8.0562 (17) Å	θ = 2.3–27.0°
*c* = 9.505 (2) Å	µ = 7.30 mm^−^^1^
α = 110.912 (3)°	*T* = 173 K
β = 96.862 (3)°	Block, colorless
γ = 95.770 (3)°	0.18 × 0.16 × 0.06 mm
*V* = 545.8 (2) Å^3^	

### Data collection

**Table:**

Bruker APEXII CCD diffractometer	2295 independent reflections
Radiation source: fine-focus sealed tube	2160 reflections with *I* > 2σ(*I*)
Graphite monochromator	*R*_int_ = 0.017
φ and ω scans	θ_max_ = 27.0°, θ_min_ = 2.3°
Absorption correction: multi-scan (*SADABS*; Sheldrick, 1996)	*h* = −9→9
*T*_min_ = 0.354, *T*_max_ = 0.669	*k* = −10→10
4550 measured reflections	*l* = −12→12

### Refinement

**Table:**

Refinement on *F*^2^	Primary atom site location: structure-invariant direct methods
Least-squares matrix: full	Secondary atom site location: difference Fourier map
*R*[*F*^2^ > 2σ(*F*^2^)] = 0.016	Hydrogen site location: inferred from neighbouring sites
*wR*(*F*^2^) = 0.038	H atoms treated by a mixture of independent and constrained refinement
*S* = 1.05	*w* = 1/[σ^2^(*F*_o_^2^) + (0.016*P*)^2^ + 0.6363*P*] where *P* = (*F*_o_^2^ + 2*F*_c_^2^)/3
2295 reflections	(Δ/σ)_max_ = 0.001
192 parameters	Δρ_max_ = 0.57 e Å^−^^3^
5 restraints	Δρ_min_ = −0.49 e Å^−^^3^

### Special details

**Table:**

Geometry. All e.s.d.'s (except the e.s.d. in the dihedral angle between two l.s. planes) are estimated using the full covariance matrix. The cell e.s.d.'s are taken into account individually in the estimation of e.s.d.'s in distances, angles and torsion angles; correlations between e.s.d.'s in cell parameters are only used when they are defined by crystal symmetry. An approximate (isotropic) treatment of cell e.s.d.'s is used for estimating e.s.d.'s involving l.s. planes.
Refinement. Refinement of *F*^2^ against ALL reflections. The weighted *R*-factor *wR* and goodness of fit *S* are based on *F*^2^, conventional *R*-factors *R* are based on *F*, with *F* set to zero for negative *F*^2^. The threshold expression of *F*^2^ > σ(*F*^2^) is used only for calculating *R*-factors(gt) *etc*. and is not relevant to the choice of reflections for refinement. *R*-factors based on *F*^2^ are statistically about twice as large as those based on *F*, and *R*- factors based on ALL data will be even larger.

### Fractional atomic coordinates and isotropic or equivalent isotropic displacement parameters (Å^2^)

**Table:**

	*x*	*y*	*z*	*U*_iso_*/*U*_eq_	
C1	0.6934 (4)	1.2898 (4)	1.1270 (3)	0.0109 (5)	
C2	0.7746 (4)	0.4547 (4)	0.2648 (3)	0.0123 (6)	
C3	0.7041 (4)	0.5062 (4)	0.3987 (3)	0.0117 (6)	
H3	0.5989	0.4387	0.4032	0.014*	
C4	0.7864 (4)	0.6576 (4)	0.5285 (3)	0.0124 (6)	
C5	0.9393 (4)	0.7518 (4)	0.5123 (3)	0.0158 (6)	
H5	0.9999	0.8543	0.5962	0.019*	
C6	0.9258 (4)	0.5562 (4)	0.2565 (4)	0.0162 (6)	
H6	0.9743	0.5245	0.1651	0.019*	
C7	0.9532 (4)	0.9560 (4)	1.0490 (3)	0.0106 (6)	
C8	0.4187 (4)	0.9302 (4)	0.4887 (3)	0.0116 (6)	
Ho1	0.647369 (16)	0.971506 (16)	0.803507 (14)	0.00854 (5)	
N1	1.0014 (3)	0.6993 (4)	0.3802 (3)	0.0168 (5)	
O1	0.7614 (3)	1.2593 (3)	1.0082 (2)	0.0162 (5)	
O2	0.4346 (3)	0.8128 (3)	0.8643 (2)	0.0136 (4)	
O3	0.7231 (3)	0.7072 (3)	0.6565 (2)	0.0150 (4)	
O4	0.7922 (3)	0.8943 (3)	1.0004 (2)	0.0133 (4)	
O5	0.9595 (3)	1.0464 (3)	0.8342 (2)	0.0123 (4)	
O6	0.4182 (3)	0.8477 (3)	0.5761 (2)	0.0161 (4)	
O7	0.7033 (3)	1.0868 (3)	0.6190 (2)	0.0135 (4)	
O8	0.4335 (3)	1.1739 (3)	0.8101 (3)	0.0197 (5)	
O9	0.3549 (5)	0.5133 (4)	0.1651 (4)	0.0462 (8)	
H1	1.102 (3)	0.760 (4)	0.373 (4)	0.011 (8)*	
H7	0.489 (6)	1.275 (4)	0.822 (6)	0.061 (17)*	
H8	0.363 (6)	1.155 (7)	0.866 (5)	0.065 (17)*	
H9	0.331 (8)	0.448 (7)	0.216 (6)	0.08 (2)*	
H10	0.269 (6)	0.501 (9)	0.096 (5)	0.09 (2)*	

### Atomic displacement parameters (Å^2^)

**Table:**

	*U*^11^	*U*^22^	*U*^33^	*U*^12^	*U*^13^	*U*^23^
C1	0.0095 (13)	0.0105 (13)	0.0124 (14)	0.0018 (10)	0.0013 (10)	0.0039 (11)
C2	0.0128 (14)	0.0109 (14)	0.0113 (14)	0.0000 (11)	0.0015 (11)	0.0027 (11)
C3	0.0087 (14)	0.0118 (14)	0.0147 (14)	0.0008 (10)	0.0034 (11)	0.0046 (11)
C4	0.0136 (14)	0.0110 (14)	0.0126 (14)	0.0038 (11)	0.0029 (11)	0.0037 (11)
C5	0.0135 (15)	0.0159 (15)	0.0141 (15)	−0.0008 (11)	0.0017 (11)	0.0020 (12)
C6	0.0149 (15)	0.0161 (15)	0.0143 (15)	−0.0008 (12)	0.0039 (11)	0.0020 (12)
C7	0.0123 (14)	0.0095 (13)	0.0088 (13)	0.0042 (11)	0.0022 (10)	0.0012 (11)
C8	0.0096 (14)	0.0128 (14)	0.0115 (14)	0.0002 (11)	0.0017 (10)	0.0038 (11)
Ho1	0.00804 (7)	0.00981 (7)	0.00735 (7)	−0.00004 (5)	−0.00004 (4)	0.00353 (5)
N1	0.0126 (13)	0.0181 (13)	0.0151 (13)	−0.0051 (10)	0.0041 (10)	0.0022 (11)
O1	0.0163 (11)	0.0151 (11)	0.0127 (10)	−0.0024 (8)	0.0061 (8)	−0.0002 (9)
O2	0.0108 (10)	0.0137 (10)	0.0141 (10)	−0.0019 (8)	−0.0005 (8)	0.0045 (8)
O3	0.0173 (11)	0.0149 (10)	0.0106 (10)	0.0019 (8)	0.0050 (8)	0.0015 (8)
O4	0.0108 (10)	0.0185 (11)	0.0109 (10)	0.0007 (8)	0.0001 (8)	0.0069 (8)
O5	0.0105 (10)	0.0159 (10)	0.0101 (10)	0.0012 (8)	0.0004 (8)	0.0052 (8)
O6	0.0170 (11)	0.0165 (11)	0.0148 (11)	−0.0054 (8)	−0.0047 (8)	0.0103 (9)
O7	0.0108 (10)	0.0189 (11)	0.0115 (10)	−0.0019 (8)	−0.0010 (8)	0.0086 (8)
O8	0.0220 (13)	0.0232 (13)	0.0201 (12)	0.0096 (10)	0.0087 (9)	0.0122 (10)
O9	0.056 (2)	0.0259 (15)	0.055 (2)	0.0023 (14)	−0.0118 (17)	0.0206 (15)

### Geometric parameters (Å, º)

**Table:**

C1—O2^i^	1.259 (4)	C8—O6	1.235 (4)
C1—O1	1.260 (4)	C8—O7^v^	1.269 (3)
C1—C2^ii^	1.507 (4)	C8—C8^v^	1.545 (6)
C2—C3	1.388 (4)	Ho1—O2	2.245 (2)
C2—C6	1.390 (4)	Ho1—O3	2.271 (2)
C2—C1^iii^	1.507 (4)	Ho1—O7	2.322 (2)
C3—C4	1.414 (4)	Ho1—O4	2.372 (2)
C3—H3	0.9500	Ho1—O5	2.399 (2)
C4—O3	1.311 (4)	Ho1—O1	2.429 (2)
C4—C5	1.400 (4)	Ho1—O8	2.435 (2)
C5—N1	1.340 (4)	Ho1—O6	2.455 (2)
C5—H5	0.9500	N1—H1	0.90 (1)
C6—N1	1.337 (4)	O8—H7	0.85 (1)
C6—H6	0.9500	O8—H8	0.85 (1)
C7—O5^iv^	1.237 (3)	O9—H9	0.85 (1)
C7—O4	1.266 (4)	O9—H10	0.85 (1)
C7—C7^iv^	1.559 (6)		
			
O2^i^—C1—O1	123.0 (3)	O4—Ho1—O5	68.67 (7)
O2^i^—C1—C2^ii^	119.6 (3)	O2—Ho1—O1	112.36 (7)
O1—C1—C2^ii^	117.4 (3)	O3—Ho1—O1	143.48 (8)
C3—C2—C6	119.6 (3)	O7—Ho1—O1	91.68 (7)
C3—C2—C1^iii^	122.1 (3)	O4—Ho1—O1	75.80 (7)
C6—C2—C1^iii^	118.3 (3)	O5—Ho1—O1	67.35 (7)
C2—C3—C4	121.0 (3)	O2—Ho1—O8	83.88 (8)
C2—C3—H3	119.5	O3—Ho1—O8	142.95 (8)
C4—C3—H3	119.5	O7—Ho1—O8	75.53 (8)
O3—C4—C5	121.8 (3)	O4—Ho1—O8	130.78 (7)
O3—C4—C3	122.0 (3)	O5—Ho1—O8	126.55 (8)
C5—C4—C3	116.2 (3)	O1—Ho1—O8	71.62 (8)
N1—C5—C4	120.9 (3)	O2—Ho1—O6	74.25 (7)
N1—C5—H5	119.5	O3—Ho1—O6	74.55 (8)
C4—C5—H5	119.5	O7—Ho1—O6	68.06 (7)
N1—C6—C2	118.5 (3)	O4—Ho1—O6	141.48 (7)
N1—C6—H6	120.7	O5—Ho1—O6	132.12 (7)
C2—C6—H6	120.7	O1—Ho1—O6	138.53 (7)
O5^iv^—C7—O4	126.2 (3)	O8—Ho1—O6	68.47 (8)
O5^iv^—C7—C7^iv^	118.1 (3)	C6—N1—C5	123.8 (3)
O4—C7—C7^iv^	115.7 (3)	C6—N1—H1	117 (2)
O6—C8—O7^v^	126.3 (3)	C5—N1—H1	119 (2)
O6—C8—C8^v^	118.0 (3)	C1—O1—Ho1	115.09 (18)
O7^v^—C8—C8^v^	115.6 (3)	C1^i^—O2—Ho1	169.7 (2)
O2—Ho1—O3	88.38 (8)	C4—O3—Ho1	132.61 (19)
O2—Ho1—O7	141.51 (7)	C7—O4—Ho1	118.19 (18)
O3—Ho1—O7	88.96 (8)	C7^iv^—O5—Ho1	116.85 (18)
O2—Ho1—O4	75.56 (7)	C8—O6—Ho1	115.09 (18)
O3—Ho1—O4	81.26 (8)	C8^v^—O7—Ho1	119.68 (18)
O7—Ho1—O4	141.63 (7)	Ho1—O8—H7	108 (4)
O2—Ho1—O5	143.17 (7)	Ho1—O8—H8	106 (4)
O3—Ho1—O5	78.02 (7)	H7—O8—H8	126 (5)
O7—Ho1—O5	73.02 (7)	H9—O9—H10	111 (6)

Symmetry codes: (i) −*x*+1, −*y*+2, −*z*+2; (ii) *x*, *y*+1, *z*+1; (iii) *x*, *y*−1, *z*−1; (iv) −*x*+2, −*y*+2, −*z*+2; (v) −*x*+1, −*y*+2, −*z*+1.

### Hydrogen-bond geometry (Å, º)

**Table:**

*D*—H···*A*	*D*—H	H···*A*	*D*···*A*	*D*—H···*A*
N1—H1···O7^vi^	0.90 (1)	1.83 (1)	2.727 (3)	171 (3)
O8—H7···O9^v^	0.85 (1)	1.95 (1)	2.787 (4)	171 (5)
O8—H8···O4^i^	0.85 (1)	1.96 (1)	2.811 (3)	177 (6)
O9—H9···O3^vii^	0.85 (1)	2.08 (1)	2.929 (4)	178 (6)
O9—H10···O1^v^	0.85 (1)	2.48 (6)	3.003 (4)	120 (5)

Symmetry codes: (i) −*x*+1, −*y*+2, −*z*+2; (v) −*x*+1, −*y*+2, −*z*+1; (vi) −*x*+2, −*y*+2, −*z*+1; (vii) −*x*+1, −*y*+1, −*z*+1.
